# Prognostic value of immune-related genes in the tumor microenvironment of lung adenocarcinoma and lung squamous cell carcinoma

**DOI:** 10.18632/aging.102871

**Published:** 2020-03-25

**Authors:** Yan Qu, Bo Cheng, Na Shao, Yibin Jia, Qingxu Song, Bingxu Tan, Jianbo Wang

**Affiliations:** 1 Department of Radiation Oncology, Qilu Hospital of Shandong University, Jinan 250012, Shandong, China; 2 Department of Radiation Oncology, Shandong Provincial Cancer Hospital, Jinan 250117, Shandong, China; 3 Department of Oncology, Shandong Provincial Hospital Affiliated to Shandong University, Jinan 250021, Shandong, China

**Keywords:** non-small cell lung cancer, tumor microenvironment, prognosis, TCGA, GEO

## Abstract

Non-small cell lung cancer (NSCLC), which consists mainly of lung adenocarcinoma (LUAD) and lung squamous cell carcinoma (LUSC), are the leading cause of cancer deaths worldwide. In this study, we performed a comprehensive analysis of the tumor microenvironmental and genetic factors to identify prognostic biomarkers for NSCLC. We evaluated the immune and stromal scores of patients with LUAD and LUSC using data from The Cancer Genome Atlas database with the ESTIMATE algorithm. Based on these scores, the differentially expressed genes were obtained and immune-related prognostic genes were identified. Functional analysis and protein-protein interaction network further revealed the immune-related biological processes in which these genes participated. Additionally, 22 subsets of tumor-infiltrating immune cells (TIICs) in the tumor microenvironment were analyzed with the CIBERSORT algorithm. Finally, we validated these valuable genes using an independent cohort from the Gene Expression Omnibus database. The associations of the immune and stromal scores with patients’ clinical characteristics and prognosis were positive in LUAD but negative in LUSC and the correlations of TIICs with clinical characteristics were clarified. Several differentially expressed genes were identified to be potential immune-related prognostic genes. This study comprehensively analyzed the tumor microenvironment and presented immune-related prognostic biomarkers for NSCLC.

## INTRODUCTION

Lung cancer is acknowledged as being the leading cause of cancer deaths worldwide, with more than 1,600,000 new cases diagnosed yearly [1]. Non-small cell lung cancer (NSCLC) is the main histological types of lung cancer, accounting for more than 85% of all lung cancer cases [2]. NSCLC is also divided into several subtypes, including lung adenocarcinoma (LUAD), lung squamous cell carcinoma (LUSC), and large-cell lung cancer (LCLC), as well as other infrequent types, among of which LUAD and LUSC are the most prevalent.

Increasing evidence indicates that the degree of malignancy of cancers is determined not only by the intrinsic features of the tumor cells, but also by components in the tumor microenvironment (TME), including immune cells, mesenchymal cells, endothelial cells, inflammatory mediators, and extracellular matrix molecules [3]. Tumor-infiltrating immune cells (TIICs) and stromal cells, which are two major types of non-tumor cell components, have been proposed to be valuable for the diagnostic and prognostic assessment of tumors [4–8]. Previous studies have suggested that tumor-infiltrating lymphocytes (TILs) have a significant effect on the clinical course of numerous cancers [9–14]. Recently, Fridman et al. [15] summarized the association of T cells with cancer clinical outcomes and found that TILs, especially cytotoxic T cells, memory T cells, and T helper 1 cells, were positively associated with good clinical outcomes in several cancers, including melanoma, head and neck, breast, bladder, urothelial, ovarian, colorectal, renal, prostate, and lung cancers. It was demonstrated that the type and density of TILs were useful for distinguishing the clinical stage, as well as the prognosis in colorectal and lung cancers [16, 17]. In addition, the TME was also reported to have an influence on the gene expression of tumor tissues and the clinical outcome [18–22]. These findings elucidated the relationship between the TME and cancer progression, raising potential methods to improve the management of malignant tumors.

Pertaining to TME, algorithms were developed to predict the tumor purity using gene expression profile data from The Cancer Genome Atlas (TCGA) database [20–22]. For example, by analyzing the specific gene expression signatures of immune and stromal cells, Yoshihara et al. created an algorithm called ESTIMATE (Estimation of STromal and Immune cells in MAlignant Tumor tissues using Expression data) to calculate immune and stromal scores for predicting the infiltration of non-tumor cells [20]. Subsequently, reports that had applied the ESTIMATE algorithm to the analyses of prostate cancer [23], breast cancer [24], and colon cancer [25] showed the effectiveness of such large-data-based algorithms, whereas its use for predicting the tumor purity of lung cancer has not been investigated in detail.

In this study, using abundant TCGA and Gene Expression Omnibus (GEO) database-sourced cases of NSCLC and the ESTIMATE algorithm [20], we explored the microenvironmental and genetic factors associated with the disease to identify immune-related prognostic biomarkers in its main subtypes including LUAD and LUSC.

## RESULTS

### Associations of immune and stromal scores with clinical characteristics and prognosis of NSCLC

**Figure 1:**
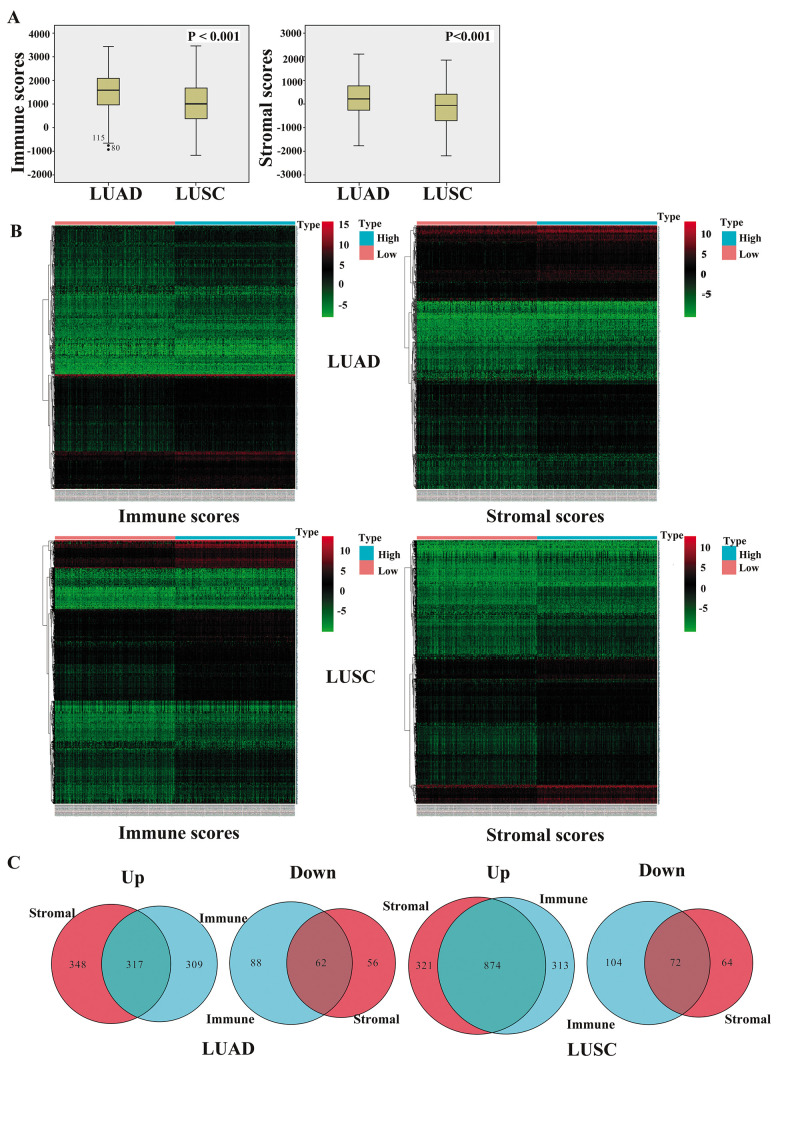
**Comparison of gene expression profiles with immune and stromal scores of NSCLC subtypes.** (**A**) The box-plot shows that there is significant difference between LUAD and LUSC at the levels of the immune scores and stromal scores (P < 0.001). (**B**) Heatmap of significantly differentially expressed genes based on immune and stromal scores. Genes with higher expression are shown in red, those with lower expression are shown in green, and those with the same expression level are in black. All results were screened at fold change > 2, P < 0.05. (**C**) Venn diagram analysis of aberrantly expressed genes based on immune and stromal scores. NSCLC, non-small cell lung cancer; LUAD, lung adenocarcinoma; LUSC, lung squamous cell carcinoma.

We downloaded TCGA-sourced gene expression profiles and clinical information of 980 cases of NSCLC, 479 (48.9%) of which were pathologically diagnosed as LUAD and 501 (51.1%) as LUSC. Of the LUAD cases, 260 (54.3%) patients were females and 219 (45.7%) were males, whereas there were 130 (25.9%) females and 371 (74.1%) males among the LUSC cases. The proportion reflected the distribution trend worldwide where females and males with LUAD had similar morbidities, while the morbidity of males with LUSC was 3 times higher than that of females with the disease. As calculated by the ESTIMATE algorithm, the immune score ranged from -942.51 to 3,442.08 among the patients with LUAD and from -1,188.16 to 3,455.61 among those with LUSC, whereas the stromal score ranged from -1,789.62 to 2,097.96 for LUAD and from -2,230.11 to 1,845.98 for LUSC (P<0.001) (Figure 1A).

**Figure 2:**
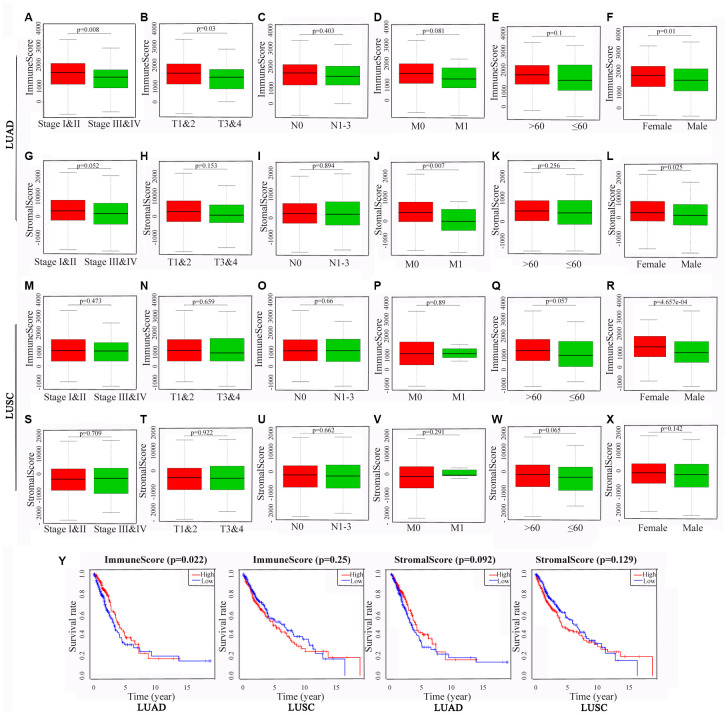
**Associations of immune and stromal scores with clinical characteristics and prognosis in NSCLC subtypes.** The LUAD and LUSC cases were respectively divided into groups with high or low median immune or stromal scores. The results represent their correlation with clinical characteristics in LUAD (**A**–**L**) and LUSC (**M**–**X**), respectively, and their correlation with overall survival in LUAD and LUSC (**Y**). P < 0.05 was used to assess differences in Log-rank test. NSCLC, non-small cell lung cancer; LUAD, lung adenocarcinoma; LUSC, lung squamous cell carcinoma.

To identify the potential correlations of the clinical characteristics with the immune and stromal scores, we divided the cases into high and low score groups according to the median scores. The statistical analyses revealed that for patients with LUAD, high immune scores were associated with an earlier clinical TNM stage (P = 0.008) and T stage (P = 0.003), and high stromal scores were associated with an earlier M stage (P = 0.007). Interestingly, both the immune and stromal scores were significantly higher for the females than those for the males (P = 0.01 and 0.025, respectively). For the patients with LUSC, none of differences in the scores were statistically significant, aside from that the immune scores being higher for the females than for the males (P < 0.001) (Figure 2A–2X).

Kaplan-Meier survival and log-rank analyses were performed to determine the potential correlations of overall survival (OS) with the immune and stromal scores (Figure 2Y). The results showed that for the patients with LUAD, high immune scores were associated with a favorable prognosis (P = 0.022), whereas the stromal scores did not have any statistically significant association with prognosis (P = 0.092). For the patients with LUSC, the OS was not significantly different for the immune or stromal scores.

### Differential gene expression analysis

To reveal the correlations of the gene expression profiles with the stromal and immune scores, we compared Affymetrix microarray data of the 479 LUAD and 501 LUSC cases obtained from TCGA, respectively. The heatmaps in Figure 1B showed distinct gene expression profiles of the cases belonging to the high and low immune and stromal score groups.

Moreover, we summarized the differentially expressed genes (DEGs) in Venn diagrams (Figure 1C). In the comparison between the LUAD groups with high and low immune scores, 626 genes were found to be upregulated and 150 genes were downregulated in the high-score group. In the comparison based on stromal scores, 665 genes were upregulated and 118 genes were downregulated in the high-score group (fold change > 2, P < 0.05). Moreover, among the DEGs, 317 genes were upregulated and 62 genes were downregulated in common in the high immune and stromal score groups compared with the gene expression in the low-score groups. Similarly, in the comparison of the LUSC cases, 1195 genes were upregulated and 136 genes were downregulated in the high stromal score group, and 1187 genes were upregulated and 176 genes were downregulated in the high immune score group (fold change > 2, P < 0.05). Additionally, 874 genes were upregulated and 72 genes were downregulated in common in the high immune and stromal score groups. We decided to focus on these DEGs for all subsequent analyses.

### Survival analysis of genes differentially expressed in common

**Figure 3:**
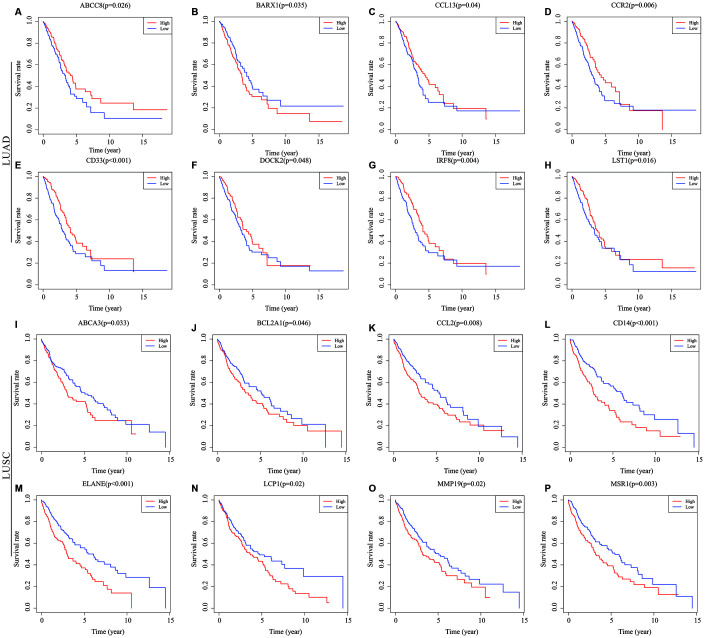
**Correlations of expression of individual immune-related DEGs in overall survival of NSCLC subtypes.** Kaplan-Meier survival curves were generated for the selected immune-related DEGs extracted DEGs extracted from the comparison of groups of high (red line) and low (blue line) gene expression. Horizontal axis: overall survival time, days; Vertical axis: survival rate. (**A**–**H**) Prognosis-related DEGs in LUAD. (**I**–**P**) Prognosis-related DEGs in LUSC. P < 0.05 was used to assess differences in Log-rank test. DEGs, differentially expressed genes; LUAD, lung adenocarcinoma; LUSC, lung squamous cell carcinoma.

To determine the potential role of the individual DEGs in OS, Kaplan-Meier survival analysis was carried out on the dataset from TCGA on the basis of the DEGs that were up-regulated and down-regulated in common in the stromal and immune score groups. Among the 379 common DEGs in LUAD, 130 were shown to significantly predict OS (P < 0.05; selected genes are shown in Figure 3A–3H). Among the 946 common DEGs in LUSC, 140 were statistically associated with OS (P < 0.05; selected genes are shown in Figure 3I–3P). All of the genes with statistically significant association with OS were listed in Supplementary Table 1.

### Functional analysis of the prognostic genes

**Figure 4:**
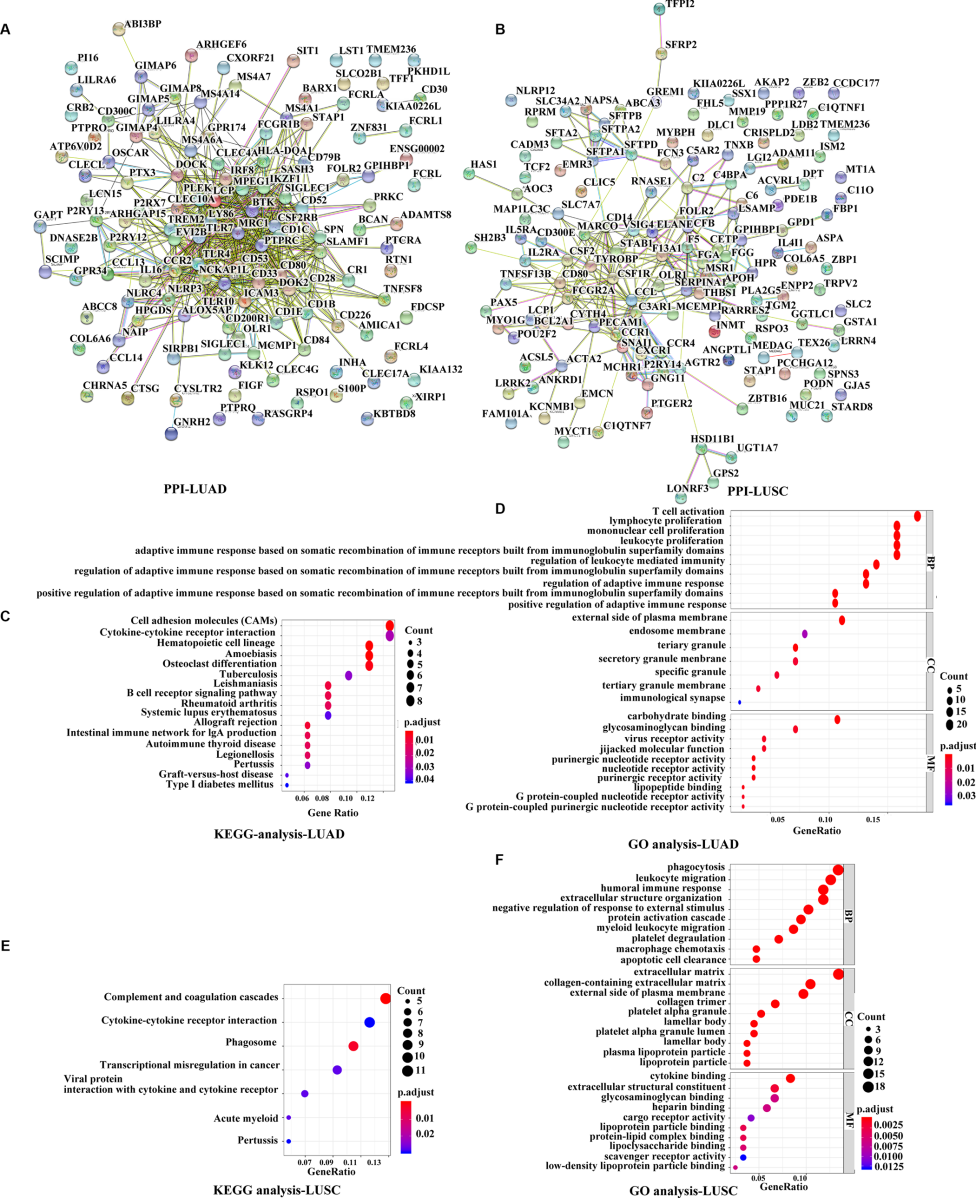
**Functional analyses of immune-related prognostic genes.** (**A**, **B**) PPI networks of the prognostic DEGs determined by the STRING database. The color of each node in the PPI network reflects the log fold-change value of the Z score of gene expression, and the size of the node indicates the number of proteins interacting with the designated protein. (**C**, **E**) KEGG analysis of immune-related prognostic genes. Top pathways with P < 0.05 and q value < 0.05 are shown. (**D**, **F**) GO analyses of the prognostic DEGs in the categories of biological processes (BP), cellular components (CC), and molecular functions (MF). PPI, Protein-protein interaction, DEGs, differentially expressed genes; GO, Gene Ontology; KEGG, Kyoto Encyclopedia of Genes and Genomes.

To further explore the interaction among the identified prognostic DEGs, we generated the protein-protein interaction (PPI) networks using the STRING tool. According to the results, the network for LUAD was made up of 101 nodes and 513 edges, where C-C motif chemokine receptor type 2 (CCR2), CD80 molecule, lymphocyte cytosolic protein 2 (LCP2), Toll-like receptor 4 (TLR4), TLR7, interleukin 10 (IL10), and protein tyrosine phosphatase receptor type C (PTPRC) were the top 7 remarkable nodes as they had the most connections with other members of the module (Figure 4A, Supplementary Figure 1A). Similarly, the network for LUSC was made up of 99 nodes and 266 edges, where complement C3a receptor 1 (C3AR1), colony-stimulating factor 1 receptor (CSF1R), C-C motif ligand 2 (CCL2), CCR1, colony-stimulating factor 2 (CSF2), CD14 molecule, and transmembrane immune signaling adaptor TYROBP were the top 7 remarkable nodes (Figure 4B, Supplementary Figure 1B). There were several immune response-critical genes in the center, including CD80, TLR4, TLR7, IL-10, CSF1R, CCL2, CCR1, CSF2, and CD14.

Consistent with the PPI network analyses, functional enrichment analyses showed that these prognostic DEGs were significantly associated with the immune status as well. The Kyoto Encyclopedia of Genes and Genomes (KEGG) analyses revealed that the DEGs were associated with several important pathways, of which the top 3 in LUAD were for cell adhesion molecules, cytokine-cytokine receptor interaction, and hematopoietic cell lineage, and the top 3 in LUSC were for complement and coagulation cascades, cytokine-cytokine receptor interaction, and phagosomes (Figure 4C, 4E). The Gene Ontology (GO) terms of the biological process, cellular component, and molecular function categories were identified respectively to be significant (P < 0.05, q value < 0.05), indicating that these genes were mainly involved in immune and inflammatory responses (Figure 4D, 4F).

### Immune landscape of the microenvironment in NSCLC

In the prior analyses, we identified that immune and stromal scores played important roles in predicting the clinical characteristics and patient prognosis in NSCLC. To better understand the immune and stromal signature, and to find more detailed relationships with LUAD and LUSC, we selected 22 available immune cell types for analyses. The selected TIICs were major cell types related to adaptive immunity [i.e., memory B cells, naïve B cells, activated memory CD4 T cells, resting memory CD4 T cells, naïve CD4 T cells, CD8 T cells, T follicular helper (Tfh) cells, gamma delta T (Tgd) cells, and regulatory T (Treg) cells] and to innate immunity [i.e., activated dendritic cells (DCs), resting DCs, eosinophils, macrophages (M0–M2), activated mast cells, resting mast cells, monocytes, activated natural killer (NK) cells, resting NK cells, neutrophils, and plasma cells].

**Figure 5:**
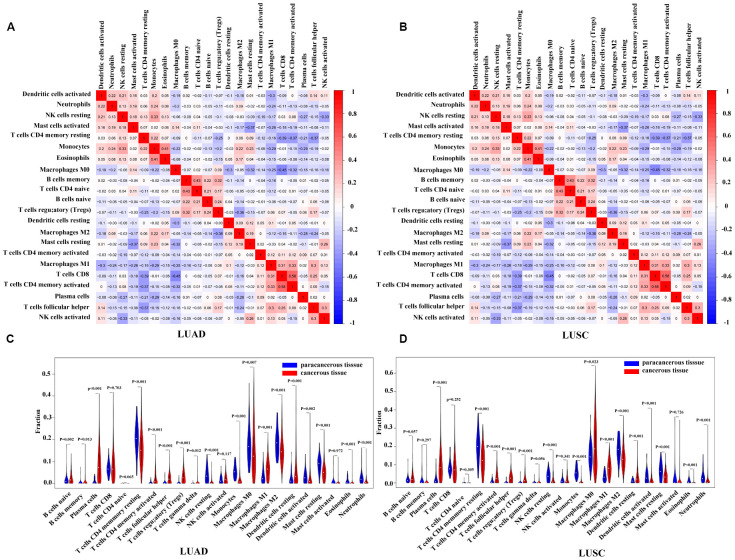
**Correlation matrix and violin diagrams of the proportions of all 22 subsets of TIICs. (A**, **B**) Correlation matrix of the correlation of the infiltration of tumor immune cells with LUAD and LUSC. (**C**, **D**) Difference of immune infiltration between cancerous tissues and paracancerous tissues. P < 0.05 was considered statistically significant. TIICs, tumor-infiltrating immune cells; LUAD, lung adenocarcinoma; LUSC, lung squamous cell carcinoma.

Using the CIBERSORT algorithm, we first evaluated the differences in the 22 TIIC subpopulations in patients with LUAD and LUSC. Supplementary Figure 2 summarized the proportions of TIICs distributed in each sample (i.e., 54 paracancerous samples and 479 patients with LUAD, and 49 paracancerous samples and 454 patients with LUSC). As shown in Figure 5A and 5B, the interrelation of the various TIICs in LUAD and LUSC varied from weak to moderate. The violin diagrams (Figure 5C, 5D) further provided visualization of the relative proportions of TIICs in all samples and the differences in their distribution between the paracancerous and cancerous samples, where the results were consistent with the heatmaps (Supplementary Figure 2) described above. The results showed the aberrant immune cell infiltration and the heterogeneity in the paracancerous and cancerous samples. In the patients with LUAD, 10 TIICs (viz., naïve B cells, memory B cells, plasma cells, activated memory CD4 T cells, Tfh cells, Treg cells, Tgd cells, resting DCs, M1 macrophages, and activated NK cells) were in a higher proportion in the cancerous tissues than those in the paracancerous tissues, whereas 9 TIICs (viz., resting memory CD4 T cells, resting NK cells, monocytes, M0 macrophages, M2 macrophages, activated DCs, resting mast cells, eosinophils, and neutrophils) were in a higher proportion in the paracancerous tissues. The results were similar in the patients with LUSC, that is, 8 TIICs (viz., plasma cells, activated memory CD4 T cells, Tfh cells, Treg cells, Tgd cells, M0 macrophages, M1 macrophages, and resting DCs) made up a higher proportion in the cancerous tissues, whereas 8 TIICs (viz., resting memory CD4 T cells, resting NK cells, monocytes, M2 macrophages, activated DCs, resting mast cells, eosinophils, and neutrophils) have a higher proportion in the paracancerous tissues (P < 0.05). Moreover, we performed a comparison of TIICs between patients with LUAD and those with LUSC. Patients with LUAD had a higher percentage of resting memory CD4^+^ T cells, Tregs, monocytes, M2 macrophages, activated NK cells, resting and activated dendritic cells, and resting mast cells than those with LUSC, while the infiltration of plasma cells, activated memory CD4+ T cells, Tfh cells, resting NK cells, M0 and M1 macrophages, and activated mast cells were lower in patients with LUAD, and other immune cells were not significantly different between patients with LUAD and those with LUSC (Supplementary Figure 3).

To further clarify the roles of these TIICs in LUAD and LUSC, we performed analyses between TIICs and T, N, M stage of the patients. As shown in Supplementary Figure 4, the results revealed that, in LUAD, memory B cells and CD8^+^ T cells were more in advanced N stage (N1-N3) than those in early N stage (N0) (P = 0.001, 0.032, respectively), whereas M0 macrophages were less in advanced N stage (P = 0.042). In LUSC, higher infiltration of Tfh cells were closely related to earlier T stage (T1-T2) (P = 0.003), while that of naïve CD4^+^ T cells were correlated with more advanced T stage (T3-T4) (P = 0.03). CD8^+^ T cells and M1 macrophages were in a higher infiltration in advanced N stage than those in early N stage (P = 0.032, 0.02, respectively). No other significant differences were observed between infiltration of TIICs and T, N, M stages.

### Validation with GEO data

**Figure 6:**
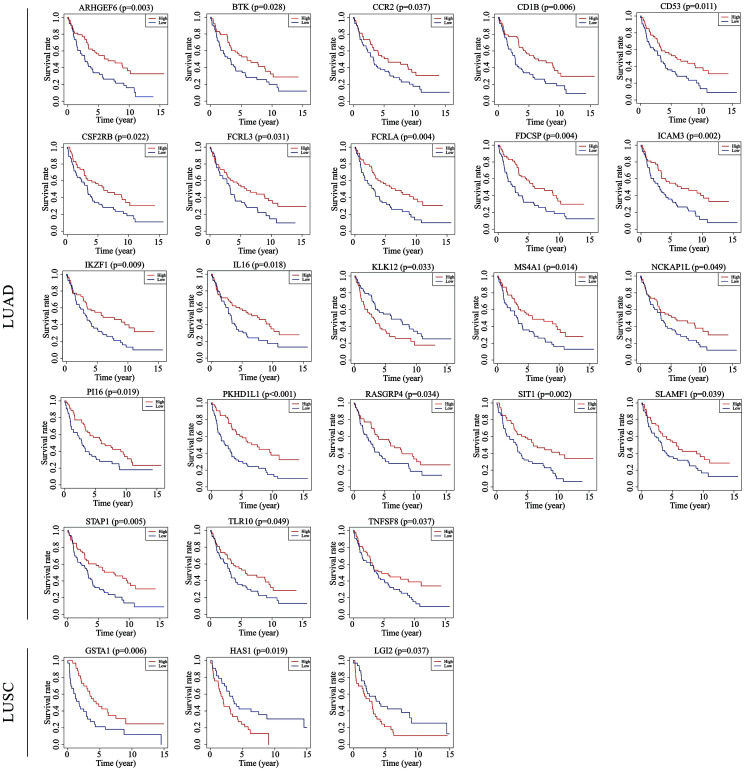
**Validation of TCGA results with other cohorts from the GEO database.** Kaplan-Meier survival curves were generated using the data of GEO database to determine the prognosis-related DEGs in TCGA. The Horizontal axis: overall survival time, days; Vertical axis: survival rate. P < 0.05 was used to assess differences in Log-rank test. GEO, Gene Expression Omnibus; TCGA, The Cancer Genome Atlas.

To verify if the prognostic value of the genes identified by TCGA analyses were critical in other cases of NSCLC, we selected an independent cohort of NSCLC cases from the GEO database (i.e., 106 LUAD cases and 66 LUSC cases, Accession Number GSE37745). Consequently, 23 of a total of 130 genes identified in the LUAD cases from TCGA were validated as being positively associated with prognosis, and 3 of the 140 genes identified in the LUSC cases were likewise validated as being prognostic (Figure 6). Among the validated genes, several were of particular interest. For example, 7 of the LUAD-associated genes [intercellular adhesion molecule 3 (ICAM3), membrane spanning 4-domains A1 (MS4A1), IL-16, Bruton tyrosine kinase (BTK), kallikrein-related peptidase 12 (KLK12), tumor necrosis factor superfamily member 8 (TNFSF8), and CCR2] had already been reported to participate in the progression of other cancers, whereas the functions of the other 16 genes have never before been reported in any cancers. In the LUSC cases, 2 [glutathione S-transferase alpha 1 (GSTA1) and hyaluronan synthase 1(HAS1)] of the 3 validated genes have been reported to play important roles in the process of cancer development, and the third gene [leucine-rich repeat LGI family member 2 (LGI2)] has never before been studied in detail.

## DISCUSSION

NSCLC is one of the most aggressive cancers worldwide, with high morbidity and lethality. Despite the progression of therapeutic methods in recent years, including chemotherapy, radiotherapy, targeted therapy, and immunotherapy, the prognosis of patients with NSCLC remain poor. One of the reasons for the dismal prognosis is a shortage of effective prognostic biomarkers for guidance on cancer therapy. Therefore, we conducted the bioinformatics analysis with TCGA to identify TME-related genes that could predict the prognosis of NSCLC patients.

Through the deconvolution of large-scale genomic data sourced from TCGA, the ESTIMATE algorithm was used to obtain immune and stromal scores to understand the microenvironment of NSCLC. For the LUAD dataset, we found that high immune scores were associated with an earlier clinical stage and T stage, and high stromal scores were positively related to an earlier M stage. In addition, higher immune scores showed close associations with better OS. For the LUSC cases, aside from the finding that the immune scores were significantly higher in the females, neither the immune nor the stromal scores were significantly associated with the clinical characteristics and prognosis. These results suggested that the microenvironment was closely associated with patients’ outcome.

In the previous study, “immune hot” tumors referred to those in which PD-L1 as well as pro- and anticancer immune cells and mediators were in a high proportion, and these tumors presented a better response to the immune treatment than others [27]. In our results, the immune scores ranged from -942.51 to 3,442.08 among the patients with LUAD and from -1,188.16 to 3,455.61 among those with LUSC. Though the average immune score of the former was significantly higher than that of the later, it did not mean that LUAD was more immune-hot than LUSC. As our results showed in Supplementary Figure 3, some immune cells were higher in LUAD (i.e., resting memory CD4^+^ T cells, Tregs, monocytes, M2 macrophages, activated NK cells, resting and activated dendritic cells, and resting mast cells), whereas some were higher in LUSC (i.e., plasma cells, activated memory CD4^+^ T cells, Tfh cells, resting NK cells, M0 and M1 macrophages, and activated mast cells), other immune cells, including the most effective immune cells, CD8^+^ T cells, were not significantly different between patients with LUAD and those with LUSC. Aside from the immune infiltration, tumors’ response to the immune treatment was also determined by the expression level of PD-L1, tumor mutation burden, EGFR mutation and other unknown factors. Therefore, apart from the analyses of the infiltration of immune cells, more analyses were required to clarify the tumors’ sensitivity to the immune treatment.

To identify the immune-related genes, we divided the samples into immune and stromal groups with either high or low scores. Common DEGs were chosen through a comparison of gene expression in a large number of LUAD and LUSC cases with high vs. low immune or stromal scores, and GO and KEGG analyses were completed based on those common DEGs. The results revealed that the DEGs played a critical role in the immunological competence of LUAD and LUSC. Thereafter, OS analyses were performed to evaluate the prognostic value of these common DEGs. Among the DEGs, 130 in LUAD and 140 in LUSC were identified to be associated with OS. PPI networks were generated to reveal the relationship and function of these prognostic genes. Most of the remarkable nodes in the modules with a high degree of connectivity (viz., CCR2, CD80 (B7-1), TLR4, TLR7, and IL-10 in LUAD; and CSF1R, CCL2, and CCR1 in LUSC) were reported to be related to proliferation, migration, invasion, and immune tolerance in NSCLC [28–34].

Next, using CIBERSORT we assessed the distinct infiltration patterns of the various immune cells in patients with LUAD and LUSC, and revealed their associations with the clinical outcomes. The proportions of most of the TIICs (viz., naïve B cells, memory B cells, plasma cells, activated memory CD4 T cells, resting memory CD4 T cells, Tfh cells, Treg cells, Tgd cells, resting DCs, activated DCs, M0 macrophages, M1 macrophages, M2 macrophages, resting NK cells, activated NK cells, monocytes, resting mast cells, eosinophils, and neutrophils in LUAD, and plasma cells, resting memory CD4 T cells, activated memory CD4 T cells, Tfh cells, Treg cells, Tgd cells, M0 macrophages, M1 macrophages, M2 macrophages, resting DCs, activated DCs, resting NK cells, monocytes, resting mast cells, eosinophils, and neutrophils in LUSC) were significantly different in the cancerous tissues compared with those in paracancerous tissues, indicating the critical role of the immune status in cancer progression in a more detailed way. Previous reports suggested that neutrophils were correlated with high-grade invasive histological subtypes of LUAD and associated with poor prognosis [35]. Consistent with this, the association between neutrophil infiltration and poor survival was also reported in gallbladder carcinoma [36]. Recent studies identified increased neutrophil infiltration in LUSC tumors [27, 37]. While in our analysis, neutrophils decreased in cancerous LUAD/LUSC compared to paracancerous LUAD/LUSC. The confliction was similar to the results of macrophages. Macrophages are generally grouped into 2 categories on the basis of their function: M1 and M2 macrophages. The M1 macrophages participate in the proinflammatory response and antitumor immunity, whereas the M2 macrophages indicate an anti-inflammatory response and pro-tumorigenic properties [38]. In the present study, we found that paracancerous LUAD/LUSC had a higher infiltration of M2 macrophages compared to cancerous LUAD/LUSC, which was inconsistant with the previous researches [27, 37]. The inconsistent results regarding to the distributions of neutrophils and M2 macrophages might due to the diverse sources of the patients between different studies. Furthermore, most studies evaluated neutrophils and M2 macrophages by immunohistochemistry-based analysis of a single or two representative surface markers, which can be misleading as many genes are expressed in different cell types. In our study, we evaluated the proportion of neutrophils and M2 macrophages by the bioinformatics tools, known as CIBERSORT. CIBERSORT is a well-recognized algorithm that can accurately estimate 22 TIICs contribution using a machine-learning support vector regression method [39–40]. As a consequence, our study put forward a novel view at the role of neutrophils and M2 macrophages in LUAD or LUSC. In addition, some of the other TIIC subpopulations, such as plasma cells, naïve B cells, and resting and activated NK cells, were also found to be associated with some clinical characteristics, reflecting the activities of the various subsets of TIICs in the development of tumors. Welsh et al. [41] showed that an increased islet/stromal mast cell ratio was an advantageous independent prognostic factor for patients with NSCLC. However, Kawai et al. [42] found no correlation of mast cells with clinical outcome, whereas G-Andre Banat et al. [43] reported that the number of mast cells was higher in tumor tissue than in tissue of healthy donors and was obviously elevated in stage III compared with stage I lung cancer. In our findings, the patients with LUAD had a higher infiltration of resting mast cells than those with LUSC, while the activated mast cells were higher in LUSC, suggesting the potential role of mast cells in the subtypes of NSCLC. Infiltrating Tfh cells were reported to play protective roles in breast cancer [44] and colorectal cancer [26], because they were positively associated with patient survival. In our results, higher infiltration of Tfh cells was closely related to earlier T stage in LUSC. The several subtypes of memory CD4^+^ T cells have various functions. For instance, they could assist on the cytoxic effect of CD8^+^ T cells, suppress harmful immunological reactions to self and foreign antigens, and even block CD8^+^ T-cell activation and NK cell killing [45, 46]. Our study reported that in LUSC, higher infiltration of naïve CD4^+^ T cells was associated with more advanced T stage, and CD8^+^ T cells and M1 macrophages were in a higher infiltration in advanced N stage. Meanwhile, in LUAD, memory B cells and CD8^+^ T cells were more whereas M0 macrophages were less in advanced N stage compared to early N stage. The above results revealed diverse roles of the immune cells in tumor progress, further study was urged to investigate role of diverse immune cells in tumor microenvironment.

Finally, we validated the prognostic genes using GEO data of an independent cohort of patients with NSCLC. Of the 23 immune-related prognostic genes identified in LUAD, 7 were reported to be associated with the progression of NSCLC or other cancers, such as ICAM3 [47], MS4A1 [48], and IL-16 [49]. The remaining 16 genes have not been previously studied in any cancers. For LUSC, only 3 immune-related genes were validated as prognostic factors. Of these, the functions of GSTA1 [50] and HAS1 [51] in lung cancer have been reported, whereas the role of LGI2 in cancer remained unknown. All these genes have the potential to become novel prognostic biomarkers for NSCLC.

However, there are potential limitations in the present study. First, this was a retrospective study based on available gene expression and clinical information data of NSCLC from TCGA and GEO database, while detailed information about resection extent, subtypes of LUAD, radiotherapy and chemotherapy were incomplete. Therefore the identification of potential prognostic genes and the roles of immune and stromal scores were limited to univariate survival analysis. When they are used for evaluating the prognosis of LUAD or LUSC patients, further independent validation with multivariate analysis using our own tissue samples with complete clinical information is warranted. Moreover, the mechanisms through which the prognostic immune-related genes modulate the initiation and progression of NSCLC requires further in vitro and in vivo investigations.

In conclusion, this study provided a comprehensive understanding of the tumor microenvironment and identified a list of immune-related prognostic genes for patients with LUAD and LUSC. Further in vivo and vitro studies are urged to investigate the exact mechanism by which these significant immune cells and genes participated in NSCLC progression in order to improve the current therapeutic practice of NSCLC.

## MATERIALS AND METHODS

### Data mining of TCGA and GEO databases

The gene expression profiles of patients with LUAD and LUSC, along with their clinical data including gender, age, clinical stage, TNM stage, histological type, survival, and outcome, were obtained directly from the data portal of TCGA (https://tcga-data.nci.nih.gov/tcga/). The procedures were carried out in accordance with the Helsinki Declaration of 1975. For data validation, the gene expression profiles of patients with NSCLC with clinical information were obtained directly from the GEO data portal (https://www.ncbi.nlm.nih.gov/geo/). The inclusion criteria were as follows: (i) patients diagnosed with LUAD or LUSC, and (ii) detection of the gene level in tissue samples. The exclusion criteria were (i) clinical data without survival times and outcomes, and (ii) datasets with small sample sizes (n < 50). Finally, the following eligible dataset was chosen: Accession Number GES37745 (n = 106 for LUAD, and n = 66 for LUSC). All the samples were chosen from patients of primary untreated tumors.

### Identification of differentially expressed genes

Data analyses were performed using the “limma” package from Bioconductor (version 3.8; https://bioconductor.org/packages/release/bioc/html/limma.html). A fold change of >2, P value of <0.05, and false discovery rate of <0.05 were set as the cutoffs for screening the DEGs.

### Survival analysis

Kaplan-Meier survival analysis was used to illustrate the correlation of the immune and stromal scores with the OS of the patients and to identify potential prognostic genes.

### Functional analysis

The PPI network was constructed from the STRING database. Functional enrichment analysis of the prognostic DEGs was performed using several packages (“colorspace,” version 1.4-1, https://cran.r-project.org/web/packages/colorspace/; “stringi,” version 1.4.3, https://cran.r-project.org/web/packages/stringi/index.html; and “ggplot2,” version 3.1.1, https://cran.r-project.org/web/packages/ggplot2/index.html), “DOSE” (version 3.8, https://bioconductor.org/packages/release/bioc/html/DOSE.html), “clusterprofiler” (version 3.8, https://bioconductor.org/packages/release/bioc/html/clusterProfiler.html), and “enrichplot” (version 3.8, http://bioconductor.org/packages/release/bioc/html/enrichplot.html) from Bioconductor (http://www.bioconductor.org/) to annotate the genes according to GO categories of biological processes, molecular functions, and cellular components. They were also used to conduct pathway enrichment analysis on the basis of data from KEGG pathways.

### Tumor microenvironment analysis

The immune and stromal scores were evaluated by applying the ESTIMATE algorithm to the downloaded LUAD and LUSC datasets, respectively. The numbers and ranges of each type of TIICs were calculated with CIBERSORT and a method developed by Bindea et al [26]. Correlation-based heatmaps were created using the package “corrplot” (version 0.84, https://cran.r-project. org/web/packages/corrplot/index.html). Venn diagrams were drawn using the package “VennDiagram” and violin plots were produced with the package “vioplot” (version 0.3.0, https://cran.r-project.org/web/packages/vioplot/index.html).

### Statistical analyses

The associations of the immune and stromal scores, TIICs, and DEGs with prognosis were analyzed by Kaplan-Meier survival analysis and the log-rank test. The correlations of the immune and stromal scores with clinical characteristics were assessed using nonparametric tests (i.e., Wilcox test if the data were divided into 2 groups; and the Kruskal test if the data were divided into 3 groups or more). Statistical analyses were performed using the SPSS 17.0 statistical software package (SPSS, Chicago, IL, USA). The differential gene expression analysis, functional analysis, and unsupervised clustering analysis were performed using R version 3.5.

### Ethics approval

Ethical approval was obtained from the Research Ethics Committee of Qilu Hospital of Shandong University as per the Helsinki declaration and its later amendments.

## Supplementary Material

Supplementary Figures

Supplementary Table 1

## References

[1] Alberg AJ, Brock MV, Ford JG, Samet JM, Spivack SD. Epidemiology of lung cancer: Diagnosis and management of lung cancer, 3rd ed: American College of Chest Physicians evidence-based clinical practice guidelines. Chest. 2013; 143:e1S–e29S. 10.1378/chest.12-2345 23649439 PMC4694610

[2] Mitchell PL, John T. Lung cancer in 2016: immunotherapy comes of age. Lancet Respir Med. 2016; 4:947–49. 10.1016/S2213-2600(16)30379-4 27890503

[3] Hanahan D, Coussens LM. Accessories to the crime: functions of cells recruited to the tumor microenvironment. Cancer Cell. 2012; 21:309–22. 10.1016/j.ccr.2012.02.022 22439926

[4] Gregório AC, Lacerda M, Figueiredo P, Simões S, Dias S, Moreira JN. Therapeutic Implications of the Molecular and Immune Landscape of Triple-Negative Breast Cancer. Pathol Oncol Res. 2018; 24:701–16. 10.1007/s12253-017-0307-2 28913723

[5] Mlecnik B, Van den Eynde M, Bindea G, Church SE, Vasaturo A, Fredriksen T, Lafontaine L, Haicheur N, Marliot F, Debetancourt D, Pairet G, Jouret-Mourin A, Gigot JF, et al. Comprehensive Intrametastatic Immune Quantification and Major Impact of Immunoscore on Survival. J Natl Cancer Inst. 2018; 110:97–108. 10.1093/jnci/djx123 28922789

[6] Zhou R, Zhang J, Zeng D, Sun H, Rong X, Shi M, Bin J, Liao Y, Liao W. Immune cell infiltration as a biomarker for the diagnosis and prognosis of stage I-III colon cancer. Cancer Immunol Immunother. 2019; 68:433–42. 10.1007/s00262-018-2289-7 30564892 PMC6426802

[7] Kang HJ, Oh JH, Chun SM, Kim D, Ryu YM, Hwang HS, Kim SY, An J, Cho EJ, Lee H, Shim JH, Sung CO, Yu E. Immunogenomic landscape of hepatocellular carcinoma with immune cell stroma and EBV-positive tumor-infiltrating lymphocytes. J Hepatol. 2019; 71:91–103. 10.1016/j.jhep.2019.03.018 30930222

[8] Jia D, Li S, Li D, Xue H, Yang D, Liu Y. Mining TCGA database for genes of prognostic value in glioblastoma microenvironment. Aging (Albany NY). 2018; 10:592–605. 10.18632/aging.101415 29676997 PMC5940130

[9] Galon J, Costes A, Sanchez-Cabo F, Kirilovsky A, Mlecnik B, Lagorce-Pagès C, Tosolini M, Camus M, Berger A, Wind P, Zinzindohoué F, Bruneval P, Cugnenc PH, et al. Type, density, and location of immune cells within human colorectal tumors predict clinical outcome. Science. 2006; 313:1960–64. 10.1126/science.1129139 17008531

[10] Johnson SK, Kerr KM, Chapman AD, Kennedy MM, King G, Cockburn JS, Jeffrey RR. Immune cell infiltrates and prognosis in primary carcinoma of the lung. Lung Cancer. 2000; 27:27–35. 10.1016/S0169-5002(99)00095-1 10672781

[11] Ropponen KM, Eskelinen MJ, Lipponen PK, Alhava E, Kosma VM. Prognostic value of tumour-infiltrating lymphocytes (TILs) in colorectal cancer. J Pathol. 1997; 182:318–24. 10.1002/(SICI)1096-9896(199707)182:3<318::AID-PATH862>3.0.CO;2-6 9349235

[12] Loi S, Drubay D, Adams S, Pruneri G, Francis PA, Lacroix-Triki M, Joensuu H, Dieci MV, Badve S, Demaria S, Gray R, Munzone E, Lemonnier J, et al. Tumor-Infiltrating Lymphocytes and Prognosis: A Pooled Individual Patient Analysis of Early-Stage Triple-Negative Breast Cancers. J Clin Oncol. 2019; 37:559–69. 10.1200/JCO.18.01010 30650045 PMC7010425

[13] Vesalainen S, Lipponen P, Talja M, Syrjänen K. Histological grade, perineural infiltration, tumour-infiltrating lymphocytes and apoptosis as determinants of long-term prognosis in prostatic adenocarcinoma. Eur J Cancer. 1994; 30A:1797–803. 10.1016/0959-8049(94)e0159-2 7880609

[14] Al-Shibli KI, Donnem T, Al-Saad S, Persson M, Bremnes RM, Busund LT. Prognostic effect of epithelial and stromal lymphocyte infiltration in non-small cell lung cancer. Clin Cancer Res. 2008; 14:5220–7. 10.1158/1078-0432.CCR-08-0133 18698040

[15] Fridman WH, Pagès F, Sautès-Fridman C, Galon J. The immune contexture in human tumours: impact on clinical outcome. Nat Rev Cancer. 2012; 12:298–306. 10.1038/nrc3245 22419253

[16] Donnem T, Kilvaer TK, Andersen S, Richardsen E, Paulsen EE, Hald SM, Al-Saad S, Brustugun OT, Helland A, Lund-Iversen M, Solberg S, Gronberg BH, Wahl SG, et al. Strategies for clinical implementation of TNM-Immunoscore in resected nonsmall-cell lung cancer. Ann Oncol. 2016; 27:225–32. 10.1093/annonc/mdv560 26578726

[17] Mlecnik B, Tosolini M, Kirilovsky A, Berger A, Bindea G, Meatchi T, Bruneval P, Trajanoski Z, Fridman WH, Pagès F, Galon J. Histopathologic-based prognostic factors of colorectal cancers are associated with the state of the local immune reaction. J Clin Oncol. 2011; 29:610–18. 10.1200/JCO.2010.30.5425 21245428

[18] Cooper LA, Gutman DA, Chisolm C, Appin C, Kong J, Rong Y, Kurc T, Van Meir EG, Saltz JH, Moreno CS, Brat DJ. The tumor microenvironment strongly impacts master transcriptional regulators and gene expression class of glioblastoma. Am J Pathol. 2012; 180:2108–19. 10.1016/j.ajpath.2012.01.040 22440258 PMC3354586

[19] Galon J, Pagès F, Marincola FM, Thurin M, Trinchieri G, Fox BA, Gajewski TF, Ascierto PA. The immune score as a new possible approach for the classification of cancer. J Transl Med. 2012; 10:1. 10.1186/1479-5876-10-1 22214470 PMC3269368

[20] Yoshihara K, Shahmoradgoli M, Martínez E, Vegesna R, Kim H, Torres-Garcia W, Treviño V, Shen H, Laird PW, Levine DA, Carter SL, Getz G, Stemke-Hale K, et al. Inferring tumour purity and stromal and immune cell admixture from expression data. Nat Commun. 2013; 4:2612. 10.1038/ncomms3612 24113773 PMC3826632

[21] Şenbabaoğlu Y, Gejman RS, Winer AG, Liu M, Van Allen EM, de Velasco G, Miao D, Ostrovnaya I, Drill E, Luna A, Weinhold N, Lee W, Manley BJ, et al. Tumor immune microenvironment characterization in clear cell renal cell carcinoma identifies prognostic and immunotherapeutically relevant messenger RNA signatures. Genome Biol. 2016; 17:231. 10.1186/s13059-016-1092-z 27855702 PMC5114739

[22] Winslow S, Lindquist KE, Edsjö A, Larsson C. The expression pattern of matrix-producing tumor stroma is of prognostic importance in breast cancer. BMC Cancer. 2016; 16:841. 10.1186/s12885-016-2864-2 27809802 PMC5095990

[23] Shah N, Wang P, Wongvipat J, Karthaus WR, Abida W, Armenia J, Rockowitz S, Drier Y, Bernstein BE, Long HW, Freedman ML, Arora VK, Zheng D, Sawyers CL. Regulation of the glucocorticoid receptor via a BET-dependent enhancer drives antiandrogen resistance in prostate cancer. eLife. 2017; 6:6. 10.7554/eLife.27861 28891793 PMC5593504

[24] Priedigkeit N, Watters RJ, Lucas PC, Basudan A, Bhargava R, Horne W, Kolls JK, Fang Z, Rosenzweig MQ, Brufsky AM, Weiss KR, Oesterreich S, Lee AV. Exome-capture RNA sequencing of decade-old breast cancers and matched decalcified bone metastases. JCI Insight. 2017; 2:95703. 10.1172/jci.insight.95703 28878133 PMC5621874

[25] Alonso MH, Aussó S, Lopez-Doriga A, Cordero D, Guinó E, Solé X, Barenys M, de Oca J, Capella G, Salazar R, Sanz-Pamplona R, Moreno V. Comprehensive analysis of copy number aberrations in microsatellite stable colon cancer in view of stromal component. Br J Cancer. 2017; 117:421–31. 10.1038/bjc.2017.208 28683472 PMC5537504

[26] Bindea G, Mlecnik B, Tosolini M, Kirilovsky A, Waldner M, Obenauf AC, Angell H, Fredriksen T, Lafontaine L, Berger A, Bruneval P, Fridman WH, Becker C, et al. Spatiotemporal dynamics of intratumoral immune cells reveal the immune landscape in human cancer. Immunity. 2013; 39:782–95. 10.1016/j.immuni.2013.10.003 24138885

[27] Domagala-Kulawik J. New Frontiers for Molecular Pathology. Front Med (Lausanne). 2019; 6:284. 10.3389/fmed.2019.00284 31867335 PMC6904313

[28] An J, Xue Y, Long M, Zhang G, Zhang J, Su H. Targeting CCR2 with its antagonist suppresses viability, motility and invasion by downregulating MMP-9 expression in non-small cell lung cancer cells. Oncotarget. 2017; 8:39230–40. 10.18632/oncotarget.16837 28424406 PMC5503609

[29] Konishi J, Yamazaki K, Azuma M, Kinoshita I, Dosaka-Akita H, Nishimura M. B7-H1 expression on non-small cell lung cancer cells and its relationship with tumor-infiltrating lymphocytes and their PD-1 expression. Clin Cancer Res. 2004; 10:5094–100. 10.1158/1078-0432.CCR-04-0428 15297412

[30] Zhan Z, Xie X, Cao H, Zhou X, Zhang XD, Fan H, Liu Z. Autophagy facilitates TLR4- and TLR3-triggered migration and invasion of lung cancer cells through the promotion of TRAF6 ubiquitination. Autophagy. 2014; 10:257–68. 10.4161/auto.27162 24321786 PMC5396095

[31] Chatterjee S, Crozet L, Damotte D, Iribarren K, Schramm C, Alifano M, Lupo A, Cherfils-Vicini J, Goc J, Katsahian S, Younes M, Dieu-Nosjean MC, Fridman WH, et al. TLR7 promotes tumor progression, chemotherapy resistance, and poor clinical outcomes in non-small cell lung cancer. Cancer Res. 2014; 74:5008–18. 10.1158/0008-5472.CAN-13-2698 25074614

[32] Vahl JM, Friedrich J, Mittler S, Trump S, Heim L, Kachler K, Balabko L, Fuhrich N, Geppert CI, Trufa DI, Sopel N, Rieker R, Sirbu H, Finotto S. Interleukin-10-regulated tumour tolerance in non-small cell lung cancer. Br J Cancer. 2017; 117:1644–55. 10.1038/bjc.2017.336 29016555 PMC5729436

[33] Yu YX, Wu HJ, Tan BX, Qiu C, Liu HZ. CSF-1R regulates non-small cell lung cancer cells dissemination through Wnt3a signaling. Am J Cancer Res. 2017; 7:2144–56. 29218239 PMC5714744

[34] Wang CL, Sun BS, Tang Y, Zhuang HQ, Cao WZ. CCR1 knockdown suppresses human non-small cell lung cancer cell invasion. J Cancer Res Clin Oncol. 2009; 135:695–701. 10.1007/s00432-008-0505-0 18972130 PMC12160165

[35] Kurebayashi Y, Emoto K, Hayashi Y, Kamiyama I, Ohtsuka T, Asamura H, Sakamoto M. Comprehensive Immune Profiling of Lung Adenocarcinomas Reveals Four Immunosubtypes with Plasma Cell Subtype a Negative Indicator. Cancer Immunol Res. 2016; 4:234–47. 10.1158/2326-6066.CIR-15-0214 26787825

[36] Zhang Y, Ma C, Wang M, Hou H, Cui L, Jiang C, Sun J, Qu X. Prognostic significance of immune cells in the tumor microenvironment and peripheral blood of gallbladder carcinoma patients. Clin Transl Oncol. 2017; 19:477–488. 10.1007/s12094-016-1553-6 27718154

[37] Mollaoglu G, Jones A, Wait SJ, Mukhopadhyay A, Jeong S, Arya R, Camolotto SA, Mosbruger TL, Stubben CJ, Conley CJ, Bhutkar A, Vahrenkamp JM, Berrett KC, et al. The Lineage-Defining Transcription Factors SOX2 and NKX2-1 Determine Lung Cancer Cell Fate and Shape the Tumor Immune Microenvironment. Immunity. 2018; 49:764–779.e9. 10.1016/j.immuni.2018.09.020 30332632 PMC6197489

[38] Chanmee T, Ontong P, Konno K, Itano N. Tumor-associated macrophages as major players in the tumor microenvironment. Cancers (Basel). 2014; 6:1670–90. 10.3390/cancers6031670 25125485 PMC4190561

[39] Barros L, Pretti MA, Chicaybam L, Abdo L, Boroni M, Bonamino MH. Immunological-based approaches for cancer therapy. Clinics (São Paulo). 2018 (Suppl 1); 73:e429s. 10.6061/clinics/2018/e429s 30133560 PMC6097086

[40] Xiong Y, Wang K, Zhou H, Peng L, You W, Fu Z. Profiles of immune infiltration in colorectal cancer and their clinical significant: A gene expression-based study. Cancer Med. 2018; 7:4496–508. 10.1002/cam4.1745 30117315 PMC6144159

[41] Welsh TJ, Green RH, Richardson D, Waller DA, O’Byrne KJ, Bradding P. Macrophage and mast-cell invasion of tumor cell islets confers a marked survival advantage in non-small-cell lung cancer. J Clin Oncol. 2005; 23:8959–67. 10.1200/JCO.2005.01.4910 16219934

[42] Kawai O, Ishii G, Kubota K, Murata Y, Naito Y, Mizuno T, Aokage K, Saijo N, Nishiwaki Y, Gemma A, Kudoh S, Ochiai A. Predominant infiltration of macrophages and CD8(+) T Cells in cancer nests is a significant predictor of survival in stage IV nonsmall cell lung cancer. Cancer. 2008; 113:1387–95. 10.1002/cncr.23712 18671239

[43] Banat GA, Tretyn A, Pullamsetti SS, Wilhelm J, Weigert A, Olesch C, Ebel K, Stiewe T, Grimminger F, Seeger W, Fink L, Savai R. Immune and Inflammatory Cell Composition of Human Lung Cancer Stroma. PLoS One. 2015; 10:e0139073. 10.1371/journal.pone.0139073 26413839 PMC4587668

[44] Gu-Trantien C, Loi S, Garaud S, Equeter C, Libin M, de Wind A, Ravoet M, Le Buanec H, Sibille C, Manfouo-Foutsop G, Veys I, Haibe-Kains B, Singhal SK, et al. CD4⁺ follicular helper T cell infiltration predicts breast cancer survival. J Clin Invest. 2013; 123:2873–92. 10.1172/JCI67428 23778140 PMC3696556

[45] Crouse J, Xu HC, Lang PA, Oxenius A. NK cells regulating T cell responses: mechanisms and outcome. Trends Immunol. 2015; 36:49–58. 10.1016/j.it.2014.11.001 25432489

[46] Rosenberg J, Huang J. CD8^+^ T Cells and NK Cells: Parallel and Complementary Soldiers of Immunotherapy. Curr Opin Chem Eng. 2018; 19:9–20. 10.1016/j.coche.2017.11.006 29623254 PMC5880541

[47] Park JK, Park SH, So K, Bae IH, Yoo YD, Um HD. ICAM-3 enhances the migratory and invasive potential of human non-small cell lung cancer cells by inducing MMP-2 and MMP-9 via Akt and CREB. Int J Oncol. 2010; 36:181–92. 10.3892/ijo_00000489 19956847

[48] Wright CM, Savarimuthu Francis SM, Tan ME, Martins MU, Winterford C, Davidson MR, Duhig EE, Clarke BE, Hayward NK, Yang IA, Bowman RV, Fong KM. MS4A1 dysregulation in asbestos-related lung squamous cell carcinoma is due to CD20 stromal lymphocyte expression. PLoS One. 2012; 7:e34943. 10.1371/journal.pone.0034943 22514692 PMC3325913

[49] Moore A, Huang WY, Danforth K, Falk R, Meade A, Bagni R, Berndt SI. Prospective evaluation of serum IL-16 and risk of prostate cancer in the Prostate, Lung, Colorectal, and Ovarian Cancer Screening Trial. Cancer Causes Control. 2018; 29:455–64. 10.1007/s10552-018-1012-5 29594819 PMC10353757

[50] Pan XD, Yang ZP, Tang QL, Peng T, Zhang ZB, Zhou SG, Wang GX, He B, Zang LQ. Expression and function of GSTA1 in lung cancer cells. Asian Pac J Cancer Prev. 2014; 15:8631–35. 10.7314/APJCP.2014.15.20.8631 25374180

[51] Sá VK, Rocha TP, Moreira A, Soares FA, Takagaki T, Carvalho L, Nicholson AG, Capelozzi VL. Hyaluronidases and hyaluronan synthases expression is inversely correlated with malignancy in lung/bronchial pre-neoplastic and neoplastic lesions, affecting prognosis. Braz J Med Biol Res. 2015; 48:1039–47. 10.1590/1414-431x20154693 26352698 PMC4671531

