# Supplementary material for: Prognostic value of immune-related genes in the tumor microenvironment of lung adenocarcinoma and lung squamous cell carcinoma
Source: Aging (Albany NY). 2020 Mar 25;12(6):4757–77. doi: 10.18632/aging.102871 (PMC7138544; doi:10.18632/aging.102871)
Supplement: Supplementary Figures [file aging-12-102871-s001.pdf]

SUPPLEMENTARY FIGURES

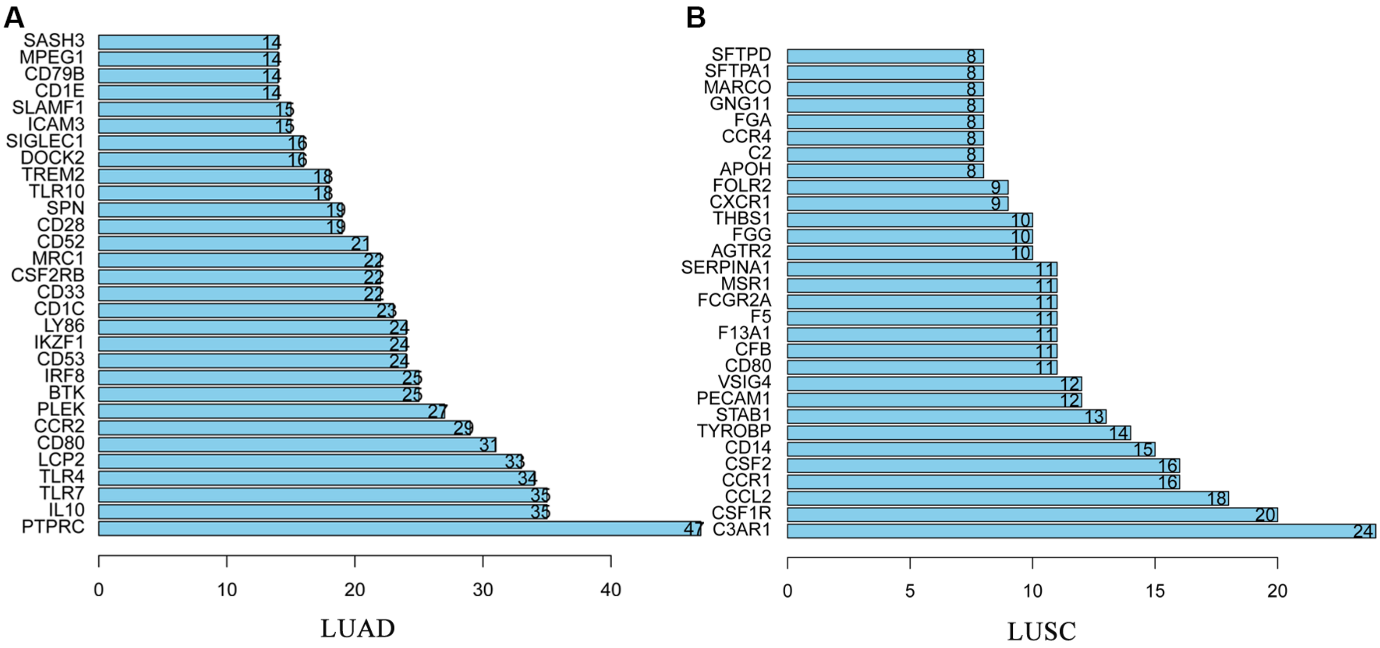

Supplementary Figure 1. Remarkable genes with a high degree of connectivity with other genes. (**A**) Top 30 remarkable genes with the most connectivity node were selected in LUAD. (**B**) Top 30 remarkable genes with the most connectivity node were selected in LUSC. LUAD, lung adenocarcinoma, LUSC, lung squamous cell carcinoma.

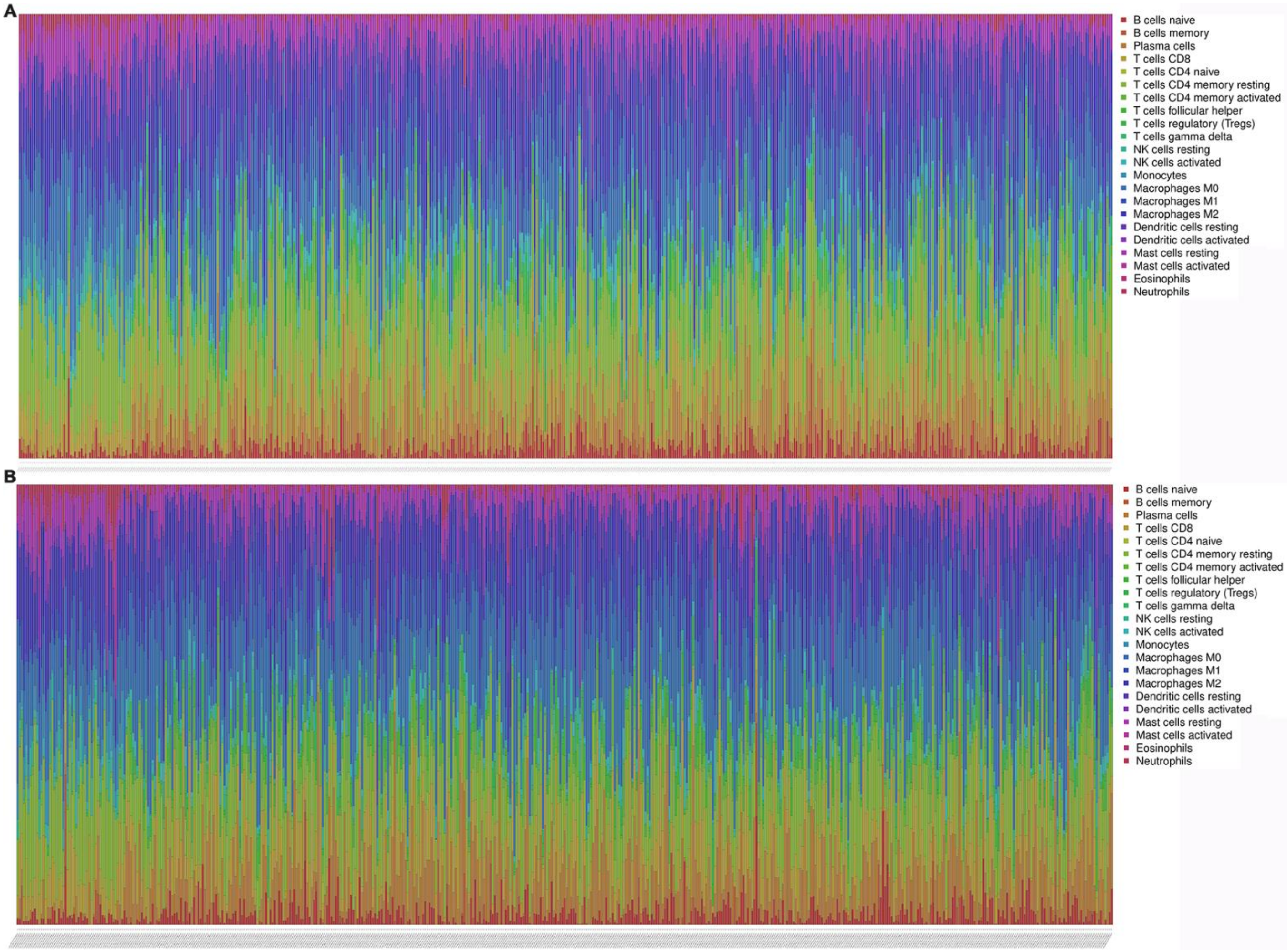

Supplementary Figure 2. Immune landscape of the microenvironment in LUAD and LUSC. Differences of 22 immune cells infiltration between cancerous tissue and paracancerous tissue in LUAD (**A**) and LUSC (**B**). Horizontal axis: list of immune cells; Vertical axis: fraction of immune cells infiltrated in samples of patients. LUAD, lung adenocarcinoma; LUSC, lung squamous cell carcinoma.

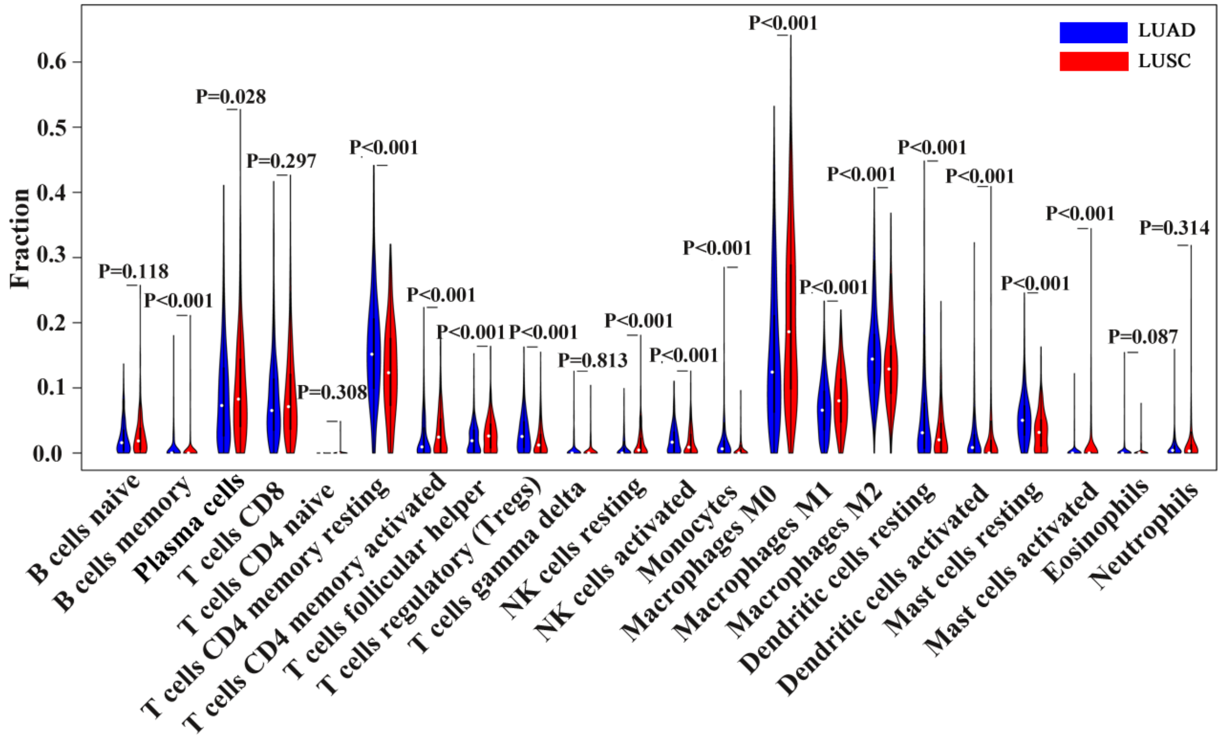

Supplementary Figure 3. Differences of immune infiltration between patients with LUAD and those with LUSC. Comparison of immune infiltration in patients with LUAD (blue) and those with LUSC (red). Horizontal axis: list of immune cells; Vertical axis: fraction of immune cells infiltrated in samples of patients. $P < 0.05$ was considered statistically significant. LUAD, lung adenocarcinoma, LUSC, lung squamous cell carcinoma.

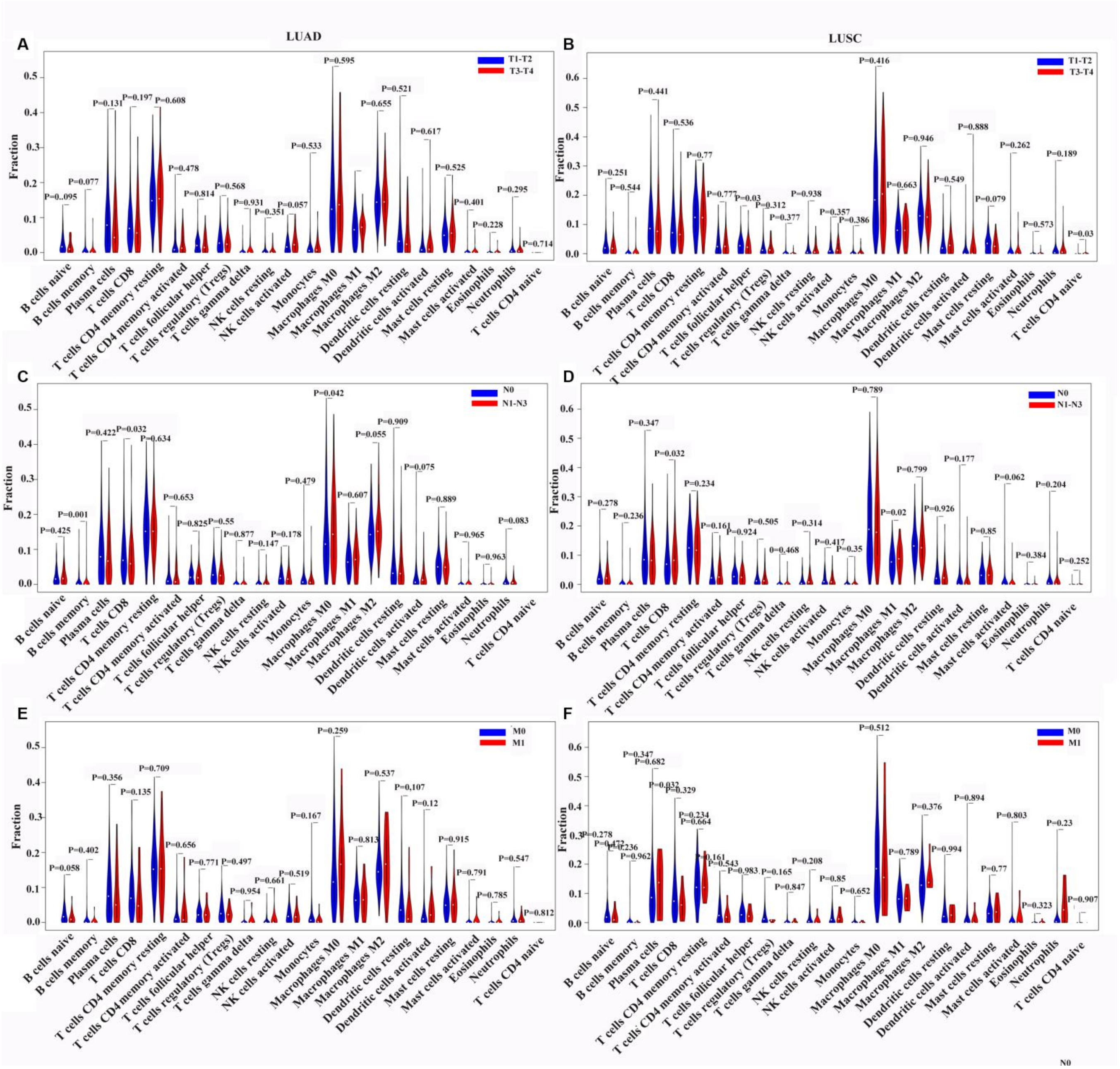

Supplementary Figure 4. Comparison of the immune infiltration and T, N, and M stages of patients with LUAD or LUSC. (**A**, **B**) Differences of immune infiltration between T1-T2 stage and T3-T4 stage in LUAD or LUSC. (**C**, **D**) Differences of immune infiltration between N0 stage and N1-N3 stage in LUAD or LUSC. (**E**, **F**) Differences of immune infiltration between M0 stage and M1 stage in LUAD or LUSC. Horizontal axis: list of immune cells; Vertical axis: fraction of immune cells infiltrated in samples of patients. P < 0.05 was considered statistically significant. LUAD, lung adenocarcinoma, LUSC, lung squamous cell carcinoma.
