# Supplementary material for: Prognostic value of immune-related genes in the tumor microenvironment of lung adenocarcinoma and lung squamous cell carcinoma
Source: Aging (Albany NY). 2020 Mar 25;12(6):4757–77. doi: 10.18632/aging.102871 (PMC7138544; doi:10.18632/aging.102871)
Supplement: Supplementary Table 1 [file aging-12-102871-s002.pdf]

SUPPLEMENTARY TABLE

Supplementary Table 1. Significant DEGs in overall survival of patients with NSCLC.

Subtype of NSCLC	Significant DEGs for overall survival
LUAD	GAPT, LILRA4, CD33, CXorf21, TMEM273, CLEC10A, CLEC17A, GPIHBP1, JAML, RUBCNL, SCIMP, BTK, TLR7, CD200R1, IRF8, CTSG, FOLR2, DOK2, TFF1, PRKCB, CHRNA5, KLK12, CD80, MPEG1, CCR2, INHA, CR1, RASGRP4, PLEK, APOC4-APOC2, RTN1, ATP6V0D2, CLEC4A, SLCO2B1, P2RY12, NLRC4, OSCAR, NAIP, ARHGAP15, P2RY13, CD53, PKHD1L1, STAP1, TNFSF8, ARHGEF6, GIMAP8, IL16, MCEMP1, CD300C, PTCRA, FCGR1B, PI16, CRB2, HLA-DQA1, LILRA6, DNASE2B, IL10, NCKAP1L, LST1, MS4A7, GIMAP4, ICAM3, LY86, SIGLEC1, PTPRQ, FCRL3, CD52, ALOX5AP, FDCSP, GIMAP5, S100P, SLAMF1, CLECL1, PTX3, RSPO1, TREM2, ZNF831, OLR1, TLR10, FCRL1, TMEM236, LCN15, FCRLA, MS4A1, CD1C, ABCC8, SPN, XIRP1, COL6A6, CSF2RB, FCRL4, CD300LG, MS4A6A, KIAA1324, CLEC4G, CD79B, GIMAP6, PTPRC, CD84, SASH3, CD1E, NLRP3, SIT1, LCP2, PTPRO, IKZF1, MRC1, CCL14, BARX1, ADAMTS8, GPR34, TLR4, GPR174, HPGDS, CD226, CCL13, CYSLTR2, MS4A14, ABI3BP, SIGLEC14, KBTBD8, CD1B, CD28, VEGFD, GNRH2, P2RX7, EVI2B, DOCK2, SIRPB1, BCAN
LUSC	CD14, ELANE, C11orf96, SNAI1, ISM2, THBS1, MYBPH, LONRF3, HPR, CCDC177, GPIHBP1, FOLR2, ACTA2, PCDHGA12, PLA2G5, CRISPLD2, VSIG4, FGA, MSR1, FGG, CFB, NLRP12, MEDAG, TFPI2, GGTLC1, FCN3, APOH, SFTA2, POU2F2, GREM1, HAS1, LGI2, UGT1A7, CCL2, FHL5, F5, HSD11B1, F13A1, TCF21, SFTPA1, FBP1, ENPP2, OLR1, SERPINA1, ZBTB16, CLIC5, SLC7A7, C2, SFTPB, MAP1LC3C, STAB1, MYO1G, AKAP2, MCHR1, CYTH4, TGM2, C3AR1, LRRK2, AOC3, RNASE1, CD300E, SFTPA2, LCP1, CSF2, MMP19, CCR1, FCGR2A, GSTA1, LRRN4, ACSL5, SLC22A31, CXCR1, TNXB, SSX1, TMEM236, C1QTNF1, TRPV2, PECAM1, ACVRL1, ZEB2, ASPA, GJA5, CADM3, STAP1, C5AR2, ADAM11, PTGER2, PDE1B, DLC1, NAPSA, MARCO, GNG11, ABCA3, SLC34A2, AGTR2, RPRM, MUC21, KCNMB1, DPT, SFRP2, STARD8, CSF1R, MCEMP1, TYROBP, PODN, SFTPD, COL6A5, RFLNA, IL2RA, LSAMP, TNFSF13B, LDB2, SPNS3, GPD1, SH2B3, MT1A, P2RY14, PPP1R27, TEX26, ANGPTL1, C4BPA, PAX5, CD80, CCR4, RUBCNL, IL5RA, G0S2, ANKRD1, EMCN, C1QTNF7, BCL2A1, C6, RARRES2, CETP, RSPO3, ZBP1, IL4I1, INMT, MYCT1, ADGRE3

Abbreviations: NSCLC, non-small cell lung cancer; LUAD, lung adenocarcinoma; LUSC, lung squamous cell carcinoma; DEGs, differentially expressed genes.
